# Complete Genome and Phylogeny of Puumala Hantavirus Isolates Circulating in France

**DOI:** 10.3390/v7102884

**Published:** 2015-10-22

**Authors:** Guillaume Castel, Mathilde Couteaudier, Frank Sauvage, Jean-Baptiste Pons, Séverine Murri, Angelina Plyusnina, Dominique Pontier, Jean-François Cosson, Alexander Plyusnin, Philippe Marianneau, Noël Tordo

**Affiliations:** 1 INRA-UMR 1062 CBGP, 755 Avenue Campus Agropolis, CS30016, 34988 Montferrier sur Lez, France; cosson@supagro.inra.fr; 2 Institut de Biologie Computationnelle, 34095 Montpellier, France; 3 INRA-UR1282, Biologie Virus Aviaire, 37380 Nouzilly, France; mathilde.couteaudier@hotmail.fr; 4 CNRS-Université Lyon 1, Laboratoire de Biométrie et Biologie Evolutive (UMR5558), F-69622 Villeurbanne, France; sauvage_frank@yahoo.fr (F.S.); jean-baptiste.pons@universite-lyon.fr (J.-B.P.); dominique.pontier@univ-lyon1.fr (D.P.); 5 LabEx ECOFECT Ecoevolutionary Dynamics of Infectious Diseases, 69622 Villeurbanne, France; 6 ANSES-Laboratoire de Lyon, Unité Virologie, 31 Avenue Tony Garnier, 69007 Lyon, France; severine.murri@anses.fr (S.M.); philippe.marianneau@anses.fr (P.M.); 7 Department of Virology, University of Helsinki, Helsinki FI-00014, Finland; alexander.plyusnin@helsinki.fi (A.P.); angelina.plyusnina@helsinki.fi (A.P.); 8 INRA-UMR Bipar, 23 Av. Général de Gaulle, 94706 Maisons-Alfort, France; 9 Institut Pasteur, Unité des Stratégies Antivirales, WHO collaborative Centre for Viral Haemorrhagic Fevers and Arboviruses, 25 rue du Docteur Roux, 75015 Paris, France; noel.tordo@pasteur.fr

**Keywords:** Hantavirus, *Puumala virus*, complete genome, phylogeny, diversity, France

## Abstract

*Puumala virus* (PUUV) is the agent of nephropathia epidemica (NE), a mild form of hemorrhagic fever with renal syndrome (HFRS) in Europe. NE incidence presents a high spatial variation throughout France, while the geographical distribution of the wild reservoir of PUUV, the bank vole, is rather continuous. A missing piece of the puzzle is the current distribution and the genetic variation of PUUV in France, which has been overlooked until now and remains poorly understood. During a population survey, from 2008 to 2011, bank voles were trapped in eight different forests of France located in areas known to be endemic for NE or in area from where no NE case has been reported until now. Bank voles were tested for immunoglobulin (Ig)G ELISA serology and two seropositive animals for each of three different areas (Ardennes, Jura and Orleans) were then subjected to laboratory analyses in order to sequence the whole S, M and L segments of PUUV. Phylogenetic analyses revealed that French PUUV isolates globally belong to the central European (CE) lineage although isolates from Ardennes are clearly distinct from those in Jura and Orleans, suggesting a different evolutionary history and origin of PUUV introduction in France. Sequence analyses revealed specific amino acid signatures along the N protein, including in PUUV from the Orleans region from where NE in humans has never been reported. The relevance of these mutations in term of pathophysiology is discussed.

## 1. Introduction

Hantaviruses are emerging zoonotic pathogens distributed worldwide except in Antarctica [1]. They may cause two severe pathologies in humans: hemorrhagic fever with renal syndrome (HFRS) and hantavirus cardiopulmonary syndrome (HCPS) [2]. Viruses of the genus *Hantavirus* are exceptions within the *Bunyaviridae* family, in being directly transmitted via aerosols ofsmall mammals (rodents, shrews, moles, and bats) excreta with no role for arthropod vectors (insects and ticks). Although a growing diversity of hantaviruses has been discovered over the last decade in insectivores and bats [3], up to now, only rodent-borne hantaviruses have been shown at the origin of human diseases. Hantaviruses are small, enveloped viruses, possessing a tri-segmented RNA genome of negative polarity: S (small) segment codes for the nucleocapsid protein and a small non-structural (NS)s protein [4] in several viral species; M (medium) segment for the glycoprotein precursor (GPC) of the envelope glycoproteins Gn and Gc; and L (large) segment for the RNA-dependent RNA polymerase (RdRp) [5]. Each hantavirus is predominantly associated with one or a few distinct mammal species and this stable virus–host association underscore the importance of host-related factors for understanding current distribution, evolution and epidemiology and mechanisms of emergence.

In Europe, zoonoses associated with hantaviruses have been recognized as a growing public health concern because of the increase of epidemics in frequency, amplitude, and geographic expansion [6]. Among hantavirus species, *Puumala* virus (PUUV) is the agent of nephropathia epidemica (NE), a mild form of HFRS in Europe. The geographical distribution of the rodent reservoir of PUUV, the bank vole (*Myodes glareolus*), is rather continuous throughout a large part of Europe. However, the spatial distribution of NE incidence is substantially smaller with a high variation at large as well as at small geographical scales [7]. The reason for this discrepancy in distribution between the reservoir and the disease is not clearly elucidated [7]. Among likely explanations, it could be that many NE cases remain undetected or misdiagnosed due to mild symptoms which possibly lead to confusion with other diseases and to a lack of clinician awareness for hantavirus diseases in non-endemic areas. Alternatively, bank voles could be free of PUUV infections in specific areas or PUUV strains that circulate in these areas would be not transmitted to or infectious for humans or provoke a milder infection. 

France offers an excellent situation to study this discrepancy since it hosts the western limit of the NE endemic region in Europe, the disease being reported only in the northeastern part, although the bank voles live almost all over the country. It is therefore important to evaluate at the country level the current distribution and the genetic characteristics of the PUUV strains circulating in bank voles.

To date, eight PUUV lineages have been described in Europe which show a strong geographical clustering [8,9] the Central European (CE) lineage (including strains from Belgium, Germany, and Slovakia) [10,11], the Alpe-Adrian (ALAD) lineage [12], the Latvian (LAT) lineage [13], the Danish (DAN) lineage [9,14], the south-Scandinavian (S-SCA) lineage [15], the north-Scandinavian (N-SCA) lineage [15,16], the Finnish (FIN) lineage [9,14] and the Russian (RUS) lineage [9]. Data are missing at the French level, only S and partial M segment sequences of viruses circulating in bank voles from the Jura region have been published, they clustered with the CE lineage. In the present study, we have performed phylogenetic analyses to specify the place of French isolates within the European PUUV phylogeny and we have looked for PUUV genetic differences between NE endemic and non-endemic areas.

Eight large forests constituted mainly by the same species of trees (beech, oak and pines) were selected. They are in areas located either in or bordering or out of the endemic zone for NE in human. Rodent trapping was performed between 2008 and 2011 to determine the global seroprevalence of PUUV in bank vole populations. We then focused on the three forests presenting the higher seroprevalence and number of positive rodents to determine the genetic characteristics of the circulating viruses: two areas where NE cases are regularly reported, *i.e.*, the French Ardennes and the Jura in the north and east of France, respectively; a third area where NE cases have never been reported, the Orleans region in the Centre of France. Two PUUV representative isolates from each of the three forests were sequenced over their full length coding regions including the L segment (6530–6562 nucleotides (nt); 2156 amino acids (aa)), the M segment (3682 nt; 1148 aa) and the S segment (1826–1830 nt; 433 aa).

Altogether, this study provides an up-to-date map of PUUV diversity in bank voles in France and improves our understanding of PUUV epidemiology and of NE incidence in different regions of France.

## 2. Materials and Methods

### 2.1. Rodent Trapping and Dissection

The trapping protocol consisted in lines of 34 and quadrats of 5 × 5 Ugglan traps separated from each other by a distance of 15 meters that were deployed for 3 successive nights. Captured rodents were weighted, sexed and blood-sampled. Blood was taken from the retro-orbital sinus and the rodent was either released or euthanized following the guidelines from Sikes *et al.* [17] for liver and lung sampling. Lungs and liver were stored in dry tubes and immediately frozen. 

All the procedures were carried out according to EC Directive 86/609/EEC for animal experiments.

### 2.2. Serological and Molecular Analysis

Serum samples were screened for previous PUUV exposure by an immunoblogulin (Ig)G ELISA on plaques coated with N recombinant protein of PUUV or controls as already described in Billecocq *et al.* [18].

Two representative isolates from each sampling areas, named PUUV/Ardennes/Mg75/2011 and PUUV/Ardennes/Mg156/2011 for Ardennes (collection date: November 2011), PUUV/Jura/Mg2/2010 and PUUV/Jura/Mg214/2010 for Jura (collection date: July 2010), and PUUV/Orleans/Mg23/2010 and PUUV/Orleans/Mg29/2010 for Orleans (collection date: June 2010) were used for the complete S, M and L sequences recovery.

RNA was extracted from lung tissue samples of positive bank voles using the TriSURE reagent (Bioline, London, UK) following the manufacturer’s recommendations. Reverse transcription-PCR (RT-PCR) was performed essentially as described earlier in Plyusnina *et al.* [19]. Sequences of primers are available upon request. PCR-amplicons were purified from agarose gel and sequenced by the Sanger method.

Complete S, M and L sequences of the six samples were deposited in GenBank database under accession numbers KT247592–KT247609 (Supplementary Table S1).

Datasets included in the study are constituted as follow.

S dataset: We used a dataset composed of the complete coding S segment sequences (nucleoprotein) of the six newly sequenced French isolates from Ardennes, Jura and Orleans and of 181 PUUV and Asian PUUV-like sequences. The five PUUV sequences (KC676609, KC676611, KC676613, KC676614, and KC676615) from Croatia, the five PUUV sequences (AB433846, AB433847, AB433848, AB433842, and AB433844) from Russia and the three PUUV sequences (EU439969, EU439971, and EU439972) from Germany, for which the regions encoding the first 3, 14 and 18 amino acid residues, respectively, are lacking, were added to the PUUV group in order to improve the representativeness of these geographic areas. The Asian PUUV-like sequences correspond to the Asian provisional species Hokkaido virus (HOKV), Muju virus (MUJV) and Fusong virus (FUSV) as reported in the 9^th^ ICTV Report [20], in order to include all known viruses with complete coding sequences for the S segments (S, open reading frame (ORF), 1293 bp).

M dataset: We used a dataset composed by the partial (2484–3010 nt) M segment sequences of the six newly sequenced French isolates and of 49 PUUV and Asian PUUV-like sequences.

L dataset: We used a dataset composed by the partial (577–989 nt) L segment sequences of the six newly sequenced French isolates and of 53 PUUV and Asian PUUV-like sequences.

All sequences were obtained from the National Institute of Allergy and Infectious Diseases (NIAID) Virus Pathogen Database and Analysis Resource (ViPR) [21,22] online through the website http://www.viprbrc.org. The list of viruses and their geographic origin are displayed in Supplementary Table S1.

Multiple sequence alignments were prepared with the Clustal Omega alignment program implemented in SEAVIEW v4.4.2 [23]. Nucleotide sequences were translated into amino acid sequences and analyzed with SEAVIEW v4.4.2. Phylogenetic reconstructions were conducted using the Maximum Likelihood (ML) approach implemented in PhyML v3.0 [24] with a statistical approximate likelihood ratio test (aLRT) of branch support, using the Asian PUUV-like species as outgroup. The optimal substitution model was identified as the GTR+G+I model (General Time Reversible) for the three segments, using a function implemented in Mega v6.0 [25]. The transition/transversion ratio was fixed to 4 and nucleotide frequencies were optimized from the data set. Rate heterogeneity was applied using discrete gamma distribution with four rate categories, and the shape parameter alpha was estimated from the data set. 

Estimate of evolutionary divergence (nucleotide and amino acid genetic diversity) within and between lineages was calculated using a function implemented in the Mega v6.0 program. Analyses were conducted using the Maximum Composite Likelihood (nucleotide) or the Poisson (amino acid) substitution model. The rate variation among sites was modeled with a gamma distribution (shape parameter = 1). All the other parameters were set to their default. 

## 3. Results

### 3.1. Seroprevalence

From 2008 to 2011, 1302 bank voles have been captured in the eight targeted French forests, of which 184 reacted positively by IgG ELISA against the recombinant PUUV N protein. The mean seroprevalence of 14.1% was, however, not homogeneously distributed (Table 1). Globally, the positive rodents were mostly found in the northeastern of France, the region endemic for NE in human, with 16% to 29.7% in Ardennes and Jura, respectively. In addition, no positive rodent was found in the southern regions that are not endemic for NE. However, 25 positive bank voles were detected among populations sampled in an area free of NE, neighboring the endemic area: the Orleans region. This region harbored a particularly high seroprevalence in bank voles (17.2%) comparable to that observed at the same period in the endemic area. About 200 of the rodent sera were systematically submitted both to serological test and to nested RT-PCR according to Bowen *et al.* [26]. As PCR positive sera were only found in a subset of the seropositive animals (data not shown), we then decided to test only the seropositive rodent sera by RT-PCR.

**Table 1:** Results of bank voles captures and seroprevalence.

Geographic Area: City (*Department*)	Date	NE Status	N° of Tested Bank Voles	N° of PUUV Seropositives	Seroprevalence (%)	N° of RT-PCR +	N° of Nested +
Charleville (*Ardennes*)	October 2008	Endemic	511	82	16.0	6 (1%)	42 (8%)
	May–June 2009						
	June 2010						
	November 2011						
Troyes (*Aube*)	May 2008	Endemic	34	5	14.7	0	1 (3%)
	October 2008						
	September 2009						
Dole (*Jura*)	Jun 2010	Endemic	229	68	29.7	9 (3.9%)	33 (14%)
Senart (*Essonne*)	May 2008	Endemic	106	4	3.8	0	1 (0.9%)
	October 2008						
	September 2009						
Orléans (*Loiret*)	June 2008	Free	145	25	17.2	1 (0.7%)	17 (12%)
	October 2008						
	April 2009						
	June–July 2010						
Fontenay-le Comte (*Vendée*)	Jun 2009	Free	19	0	0		
Meillers (*Allier*)	Jun 2008	Free	227	0	0		
	Sept 2008						
	Apr 2009						
	May 2009						
Bourg en Bresse (*Ain*)	Mar 2008	Free	31	0	0		
	May 2008						
	Sept 2009						
*TOTAL*			*1302*	*184*	*14.1*	*16 (1.2%)*	*94 (7.2%)*

NE: Nephropathia Epidemica; PUUV: *Puumala* virus; RT-PCR: reverse transcription-PCR.

### 3.2. Phylogenetic Analyses

In order to elucidate if genetic differences in the PUUV viruses circulating in the different regions may explain the discrepancy in human transmission, we have compared viral isolates representative of the Orleans non-endemic region to those selected in the two main endemic areas of Charleville and Dole. This corresponds to the first description of the complete genome coding sequences of PUUV strains circulating in bank vole populations in France. Until now, only S and partial M segment sequences from PUUV circulating in the Jura region have been published [27]. Complete coding sequences of the three genomic segments of two PUUV isolates for each of the three areas presenting the higher seroprevalence and number of positive rodents were determined (See Supplementary Table S1 for their GenBank accession numbers).

**Figure 1:**
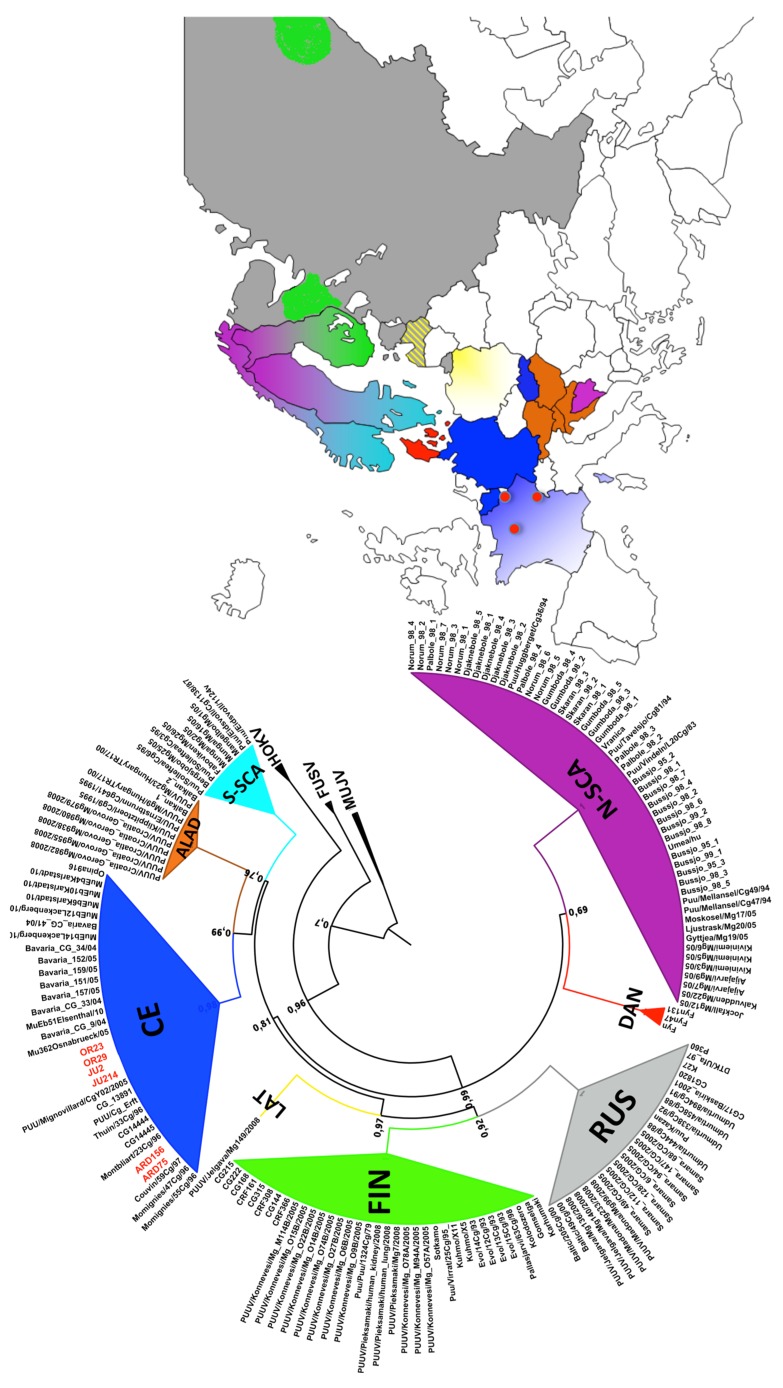
Circular phylo-tree based on the complete N protein coding sequences (the S segment N-open reading frame (ORF)). The same colors are used for the known Puumala virus (PUUV) lineages and their respective countries of origin on the map. French PUUV from this study are highlighted in red on the tree and red points on the map indicate their geographic localizations.

Phylogenetic analysis of the complete S coding sequences showed, as expected, that the French PUUV isolates cluster among the CE lineage (Figure 1) (bootstrap support value of 99%).

**Figure 2:**
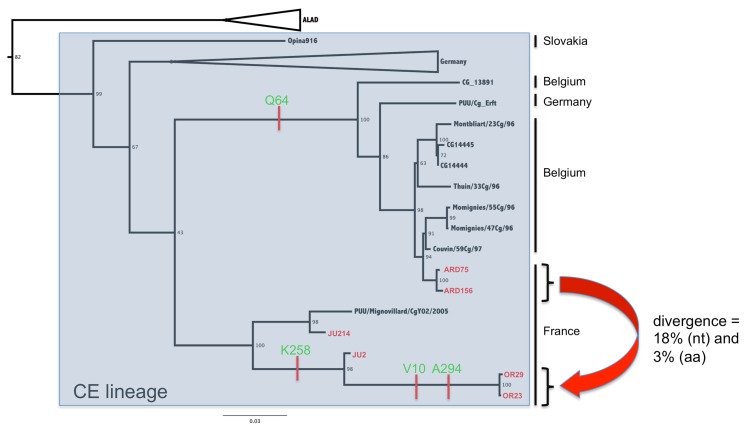
Phylogenetic tree based on the complete N protein coding sequences (the S segment N- ORF) of PUUV of the central European (CE) lineage. Scale bar represent the average number of substitutions per site. French isolates from this study are in red and specific amino acid (aa) signatures are indicated on the branches of the tree.

This is highlighted in Figure 2 that focused on the CE lineage that also included the Belgian, German and Slovakian isolates. Interestingly, isolates from Ardennes are more closely related to the Belgian isolates, which appear to be logical considering their geographic proximity. However, isolates from Jura/Orleans occupied an outsider’s position without being in close proximity to each other: while the two Orleans isolates are very similar, those from Jura are distinct, one segregating closer to the isolate from MignovillardY02 previously published [27], the other being more related to the Orleans isolates. 

**Figure 3:**
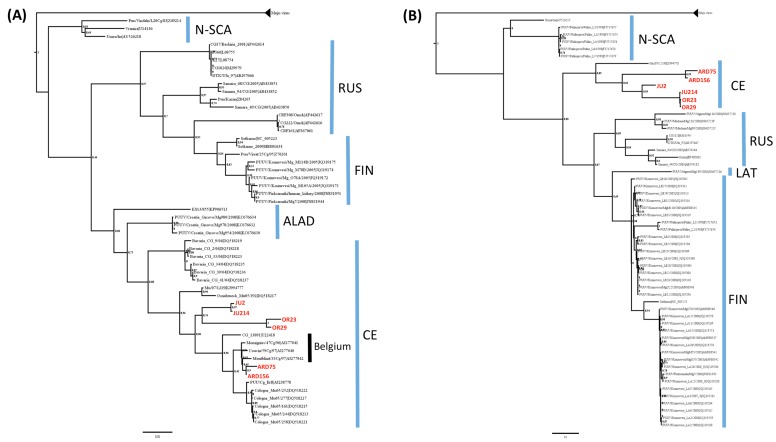
Phylogenetic trees based (**A**) on the partial (2484–3010 nt) M segment sequences and (**B**) on the partial (577–989 nt) L segment sequences of PUUV. Scale bar represent the average number of substitutions per site. French isolates are in red. Blue bars indicate known PUUV lineages.

Phylogenetic analyses of the partial M and L segment sequences confirm this result (Figure 3). Although all the lineages are not represented because of a lack of sequences, French isolates clustered in both phylogenies among the CE lineage, with a clear division between isolates from Ardennes and isolates from Orleans and Jura. Similarly to the S segment, the M segment analysis shows that isolates from French Ardennes are very close to strains from Belgian Ardennes.

### 3.3. Genetic Diversity of French Isolates

Analyses of the complete coding sequence estimate that the evolutionary divergence between the PUUV lineages in France reaches up to 18% at the nucleotide level and 3% at the amino acid level for the S segment (Figure 2 and Table 2). This divergence is almost the same for the other segments with a global tendency to slightly more divergence for the M segments (19% nt and 4% aa) and slightly less divergence for the L segment (15% nt and 3% aa). As already documented for PUUV [28,29] or other hantaviruses [30], only a few non-synonymous substitutions are observed, the majority of mutations are silent, suggesting strong purifying selection.

**Table 2:** Evolutionary divergence between French PUUV isolates nucleotide sequence (**A**) and amino acid sequence (**B**) levels. The results are expressed in percentage (%) of divergence. *: not tested.

**(A)**	**Nucleotide**																	
	**S segment**						**M segment**						**L segment**					
	**1**	**2**	**3**	**4**	**5**	**6**	**1**	**2**	**3**	**4**	**5**	**6**	**1**	**2**	**3**	**4**	**5**	**6**
**(1) Ard156**	*						*						*					
**(2) Ard75**	0	*					1	*					2	*				
**(3) OR23**	17	17	*				19	18	*				14	15	*			
**(4) OR29**	18	17	0	*			18	17	2	*			14	15	1	*		
**(5) JU2**	16	15	7	7	*		14	14	17	16	*		12	12	10	10	*	
**(6) JU214**	16	15	15	15	7	*	14	14	17	15	1	*	12	11	9	9	3	*
**(B)**	**Amino acid**																	
	**S segment**						**M segment**						**L segment**					
	**1**	**2**	**3**	**4**	**5**	**6**	**1**	**2**	**3**	**4**	**5**	**6**	**1**	**2**	**3**	**4**	**5**	**6**
**(1) Ard156**	*						*						*					
**(2) Ard75**	0	*					0	*					1	*				
**(3) OR23**	3	3	*				3	3	*				3	3	*			
**(4) OR29**	3	3	0	*			3	3	1	*			2	3	0	*		
**(5) JU2**	2	2	2	2	*		2	2	4	3	*		2	2	2	2	*	
**(6) JU214**	1	1	2	2	1		2	3	4	3	0	*	2	3	2	2	1	*

### 3.4. Amino Acid Specific Signatures

**Figure 4:**
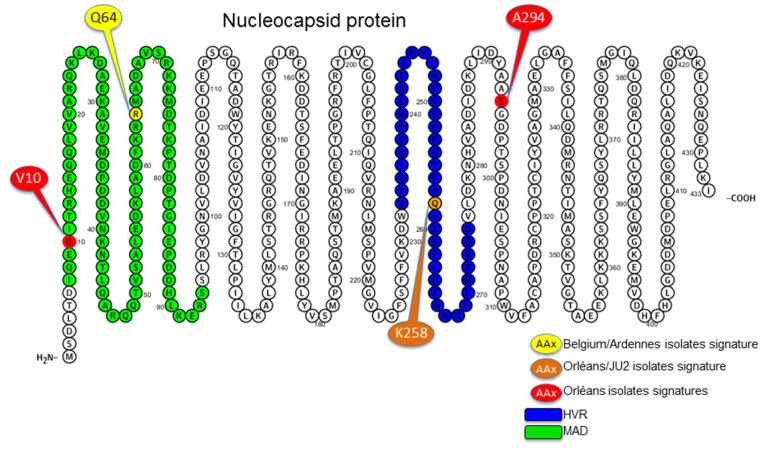
PUUV nucleocapsid protein aa sequence visualized with Protter—visualize proteoforms [31]. The used aa sequence corresponds to the consensus sequence of the S sequences dataset. The major antigenic domain (MAD) is represented in green and the hyper variable region (HVR) in blue.

All genetic lineages of PUUV possess specific amino acid “signatures” [9]. Concerning the French isolates, it is of note that within the CE lineage, only those from Ardennes possess the Q64 signature characteristic of the Belgian strains [9], outlining the close relationship between them. This residue is embedded in the major antigenic site of the N protein. On the other hand, sequences from Orleans have two specific amino acid residues (V10 within the major antigenic site and A294) at very conserved positions and share another one with the JU2 Jura isolate, K258 located in the hyper variable region of the protein (Figure 4).

## 4. Discussion

The distribution and genetic variability of PUUV circulating in France has been overlooked until now and remains poorly understood. During a population survey from 2008 to 2011, we trapped 1302 bank voles in eight different forests of France located in four regions known to be endemic for NE and four regions from where no NE case has been reported until now. Surprisingly, rodents from one forest located outside the endemic area, in the Orleans region, harbored a high PUUV seroprevalence (17.2%) comparable to that observed in the endemic area. To explore if genetic characteristics of the corresponding viruses may explain this discrepancy in human transmission, we have compared the complete genome coding sequences of two PUUV isolates for the non-endemic Orleans region with two PUUV isolates for each of two areas where NE cases are most regularly reported, the French Ardennes and the Jura. This study constitutes the first molecular description of the complete genome of French PUUV isolates and the first description of the French PUUV isolates diversity between endemic and non-endemic areas. 

Phylogenetic analyses using N, M and L coding regions revealed that French PUUV isolates belong logically to the CE lineage. However, strains from Ardennes are significantly divergent from those from Jura and Orleans. Our results suggest two separate introductions of PUUV (from a hypothetical “German ancestor”) to what is currently the territory of France: first, from what is currently Belgium, establishing the Ardennes sublineage, very closely related to the Belgium strains circulating just across the French–Belgian border [11]. The members of this sublineage are sharing their specific Q64 amino acid signature embedded in the major antigenic domain of the N protein [9]. The other sublineage is more spread across a central area in France, from east (Jura) to central (Orleans). It is more divergent and may originate directly from what is currently Germany or from central European areas. It also presents specific amino acid signatures more dispersed along the N protein, from the major antigenic domain at the *N*-terminus to the central hyper-variable region. An interesting point concerns the two isolates from the Dole forest (JU2, JU214) that behave differently in phylogenies realized with the different gene segments. Using M segment they are closely related and distinct from the Orleans isolates (OR23, OR29). Using S segment, JU2 is closer from the OR isolates, while using L segment, JU214 is closer from them. How this observation would suggest a possible reassortment between segments remains unclear and must be verified by increasing the number of sequences to be compared. 

A microevolution process with accumulation of point mutations at a local geographic scale resulting in the generation of few preferred genotypes [28,29], could constitute a second hypothesis to explain this co-circulation of PUUV strains in France.

Sequence analyses revealed specific amino acid signature of the Orleans region, from where NE in humans has never been reported. Three of these residues are located within the major antigenic domain [32,33,34] or within the hyper variable region [9] both carrying antigenic epitopes. It would be interesting to investigate if the point mutation V10D in the main antigenic of N, specific to the Orleans isolates, results in modification in antigenicity. This may induce a stronger immunogenicity in humans leading to a better virus clearance explaining that no human case is observed in the Orleans area. The inverse hypothesis may be formulated, based on the observation that for many viruses, pathogenicity is linked to a disproportioned innate immune response able to induce damages to the host [35] what may be the reason of an immune mediated pathogenesis in hantavirus infection [36]. In this case, the V10D mutation could be responsible for a lower activation of the innate immune system and thus reduced pathogenic infection in humans. It would be important to clarify whether these subtle differences between genetically closely related hantaviruses may induce very different virulence. Currently, no reverse genetic system is available for hantaviruses to investigate this point and only comparative genomics may help to understand the determinants of excretion, transmission and human pathogenicity [37]. Alternatively to these hypotheses about strain virulence or immunogenicity, different human exposure due to rodent dynamics or human behavior may also contribute to the efficiency or the opportunity of virus transmission [38,39].

The recent discovery of NE cases far outside the reported area for NE distribution in France [40] emphasizes the need for a better assessment of PUUV distribution and diversity in a country that limit the endemic area at the western part of Europe. Moreover, further phylogeographic analyses are needed to investigate the dynamics and evolutionary history of viral populations of PUUV at the European level and notably to reconstruct the precise origins of the French lineages. At the present stage, insufficient geographical sampling could influence phylogeographic interpretations [41,42]. Many areas/countries are still suffering a lack of sufficient data, only based on very short sequences, and there is a crucial need to develop an adequate sampling coverage for studying the genetic variation of PUUV in France and in Europe. This would allow better statistical evaluations of future phylogeographical hypotheses [42].

## Supplementary Files

Supplementary File 1
