# Supplementary material for: Complete Genome and Phylogeny of Puumala Hantavirus Isolates Circulating in France
Source: Viruses. 2015 Oct 22;7(10):5476–88. doi: 10.3390/v7102884 (PMC4632392; doi:10.3390/v7102884)
Supplement: Supplementary File 1 [file viruses-07-02884-s001.pdf]

viruses

Supplementary Materials

Table S1. PUUV sequences used in the study

Strain Name	GenBank Accession No (S segment)	GenBank Accession No (M segment)	GenBank Accession No (L segment)	Sampling Date	GenBank Host	Country	Lineage
PUU/Ernstbrunn/Cg641/1995	AJ888752	-	-	1995	Myodes glareolus	Austria	
PUU/Klippitztoerl/Cg9/1995	AJ888751	-	-	1995	Myodes glareolus	Austria	
PUUV/Croatia_Gerovo/Mg938/2008	KC676609	-	-	2008	Myodes glareolus	Croatia	
PUUV/Croatia_Gerovo/Mg955/2008	KC676611	-	-	2008	Myodes glareolus	Croatia	
PUUV/Croatia_Gerovo/Mg954/2008	-	KC676630	-	2008	Myodes glareolus	Croatia	
PUUV/Croatia_Gerovo/Mg978/2008	-	KC676632	-	2008	Myodes glareolus	Croatia	
PUUV/Croatia_Gerovo/Mg979/2008	KC676613	-	-	2008	Myodes glareolus	Croatia	ALAD
PUUV/Croatia_Gerovo/Mg980/2008	KC676614	KC676634	-	2008	Myodes glareolus	Croatia	
PUUV/Croatia_Gerovo/Mg982/2008	KC676615	-	-	2008	Myodes glareolus	Croatia	
PUUV/Mg23/HungaryTR17/00	FN377822	-	-	2000	Myodes glareolus	Hungary	
PUUV/Mg9/HungaryTR17/00	FN377821	-	-	2000	Myodes glareolus	Hungary	
Balkan_1	AJ314600	-	-	?	Myodes glareolus	Balkan	
Balkan_2	AJ314601	-	-	?	Myodes glareolus	Balkan	
Bavaria 151/05	EU439968	-	-	2005	Myodes glareolus	Germany	
Bavaria 152/05	EU439969	-	-	2005	Myodes glareolus	Germany	
Bavaria 157/05	EU439971	-	-	2005	Myodes glareolus	Germany	CE
Bavaria 159/05	EU439972	-	-	2005	Myodes glareolus	Germany	
Bavaria CG 33/04	DQ016430	DQ518223	-	2004	Myodes glareolus	Germany	
Bavaria CG 34/04	AY954723	DQ518235	-	2004	Myodes	Germany	

Strain Name	GenBank Accession No (S segment)	GenBank Accession No (M segment)	GenBank Accession No (L segment)	Sampling Date	GenBank Host	Country	Lineage
					glareolus		
Bavaria CG 39/04	-	DQ518236	-	2004	Myodes glareolus	Germany	
Bavaria CG 41/04	DQ016432	DQ518237	-	2004	Myodes glareolus	Germany	
Bavaria CG 9/04	AY954722	DQ518219	-	2004	Myodes glareolus	Germany	
Bavaria CG 2/04	-	DQ518218	-	2004	Myodes glareolus	Germany	
PUU/Cg_Erft	-	AJ238778	-	?	Myodes glareolus	Germany	
CG 13891	U22423	U22418	-	1985	Myodes glareolus	Belgium	
CG14444	AJ277075	-	-	1985	Myodes glareolus	Belgium	
CG14445	AJ277076	-	-	1985	Myodes glareolus	Belgium	
Couvin/59Cg/97	AJ277034	AJ277040	-	1997	Myodes glareolus	Belgium	
Momignies/47Cg/96	AJ277032	AJ277041	-	1996	Myodes glareolus	Belgium	
Momignies/55Cg/96	AJ277033	-	-	1996	Myodes glareolus	Belgium	
Montbliart/23Cg/96	AJ277031	AJ277042	-	1996	Myodes glareolus	Belgium	
Mu362Osnabrueck/05	JN696358	-	-	2005	Myodes glareolus	Germany	
Osnabrueck_Mu05/392	-	DQ518217	-	2005	Myodes glareolus	Germany	
MuEb10Karlstadt/10	JN696373	-	-	2010	Myodes glareolus	Germany	
MuEb12Lackenberg/10	JN696374	-	-	2010	Myodes glareolus	Germany	
MuEb14Lackenberg/10	JN696375	-	-	2010	Myodes glareolus	Germany	
MuEb4Karlstadt/10	JN696372	-	-	2010	Myodes glareolus	Germany	
MuEb51Elsenthal/10	JN696376	-	-	2010	Myodes glareolus	Germany	
Cologne_Mu05/161	-	DQ518215	-	2005	Myodes glareolus	Germany	

Strain Name	GenBank Accession No (S segment)	GenBank Accession No (M segment)	GenBank Accession No (L segment)	Sampling Date	GenBank Host	Country	Lineage
Cologne_Mu05/244	-	DQ518213	-	2005	Myodes glareolus	Germany	
Cologne_Mu05/252	-	DQ518222	-	2005	Myodes glareolus	Germany	
Cologne_Mu05/258	-	DQ518221	-	2005	Myodes glareolus	Germany	
Cologne_Mu05/277	-	DQ518227	-	2005	Myodes glareolus	Germany	
MuEb6Karlstadt/10	JN696371	-	-	2010	Myodes glareolus	Germany	
Mu/07/1219	KJ994776	KJ994777	KJ994778	2007	Myodes glareolus	Germany	
Opina916	AF294652	-	-	1996	Myodes glareolus	Slovakia	
PUU/Mignovillard/CgY02/2005	AM695638	-	-	2005	Myodes glareolus	France	
Thuin/33Cg/96	AJ277030	-	-	1996	Myodes glareolus	Belgium	
ARD156	KT247592	KT247603	KT247609	2011	Myodes glareolus	France	
ARD75	KT247593	KT247602	KT247608	2011	Myodes glareolus	France	
JU2	KT247596	KT247598	KT247606	2010	Myodes glareolus	France	
JU214	KT247597	KT247599	KT247607	2010	Myodes glareolus	France	
OR23	KT247594	KT247600	KT247604	2010	Myodes glareolus	France	
OR29	KT247595	KT247601	KT247605	2010	Myodes glareolus	France	
Fyn	AJ238791	-	-	1990	Myodes glareolus	Denmark	
Fyn47	AJ278092	-	-	2000	Myodes glareolus	Denmark	DAN
Fyn131	AJ278093	-	-	2000	Myodes glareolus	Denmark	
CG144	AF367064	-	-	1999-2000	Myodes glareolus	Russia	
CG168	AF367065	-	-	1999-2000	Myodes glareolus	Russia	FIN
CG215	AF367066	-	-	1999-2000	Myodes	Russia	

Strain Name	GenBank Accession No (S segment)	GenBank Accession No (M segment)	GenBank Accession No (L segment)	Sampling Date	GenBank Host	Country	Lineage
					glareolus		
CG222	AF367067	AF442616	-	1999-2000	Myodes glareolus	Russia	
CG315	AF367068	-	-	1999-2000	Myodes glareolus	Russia	
CRF161	AF367069	AF367061	-	1999-2000	Myodes rufocanus	Russia	
CRF308	AF367070	AF442617	-	1999-2000	Myodes rufocanus	Russia	
CRF366	AF367071	-	-	1999-2000	Myodes rufocanus	Russia	
Evo/12Cg/93	Z30702	-	-	1993	Myodes glareolus	Finland	
Evo/13Cg/93	Z30703	-	-	1993	Myodes glareolus	Finland	
Evo/14Cg/93	Z30704	-	-	1993	Myodes glareolus	Finland	
Evo/15Cg/93	Z30705	-	-	1993	Myodes glareolus	Finland	
Gomselga	AJ238790	-	-	1995	Myodes glareolus	Russia	
Karhumaki	AJ238788	-	-	1995	Myodes glareolus	Russia	
Kolodozero	AJ238789	-	-	2005	Myodes glareolus	Russia	
Kuhmo/X11	GU808825	-	-	2000	Myodes glareolus	Finland	
Kuhmo/X5	GU808824	-	-	2000	Myodes glareolus	Finland	
PUUV/Pallasjarvi/Pallas_L1/1998	-	-	FJ717673	1998	Myodes glareolus	Finland	
PUUV/Pallasjarvi/Pallas_L2/1998	-	-	FJ717674	1998	Myodes glareolus	Finland	
Pallasjarvi/63Cg/98	AJ314597	-	-	1998	Myodes glareolus	Finland	
Puu/Puu/1324Cg/79	Z46942	-	-	1979	Myodes glareolus	Finland	
Puu/Virrat/25Cg/95)	Z69985	Z70201	-	1995	Myodes glareolus	Finland	
PUUV/Konnevesi_LA1/2008	-	-	JQ319259	2008	Myodes glareolus	Finland	

Strain Name	GenBank Accession No (S segment)	GenBank Accession No (M segment)	GenBank Accession No (L segment)	Sampling Date	GenBank Host	Country	Lineage
PUUV/Konnevesi_LA11/2008	-	-	JQ319269	2008	Myodes glareolus	Finland	
PUUV/Konnevesi_LA12/2008	-	-	JQ319270	2008	Myodes glareolus	Finland	
PUUV/Konnevesi_LA15/2008	-	-	JQ319273	2008	Myodes glareolus	Finland	
PUUV/Konnevesi_LA2/2008	-	-	JQ319260	2008	Myodes glareolus	Finland	
PUUV/Konnevesi_LA20/2005	-	-	JQ319278	2005	Myodes glareolus	Finland	
PUUV/Konnevesi_LA22/2008_9	-	-	JQ319280	2008	Myodes glareolus	Finland	
PUUV/Konnevesi_LA26/2008_9	-	-	JQ319284	2008	Myodes glareolus	Finland	
PUUV/Konnevesi_LA3/2007_9	-	-	JQ319261	2007	Myodes glareolus	Finland	
PUUV/Konnevesi_LA4/2009	-	-	JQ319262	2009	Myodes glareolus	Finland	
PUUV/Konnevesi_LA6/2009	-	-	JQ319264	2009	Myodes glareolus	Finland	
PUUV/Konnevesi_LA7/2009	-	-	JQ319265	2009	Myodes glareolus	Finland	
PUUV/Konnevesi_LB1/2008	-	-	JQ319285	2008	Myodes glareolus	Finland	
PUUV/Konnevesi_LB12/2008	-	-	JQ319296	2008	Myodes glareolus	Finland	
PUUV/Konnevesi_LB16/2008_9	-	-	JQ319300	2008	Myodes glareolus	Finland	
PUUV/Konnevesi_LB18/2005	-	-	JQ319302	2005	Myodes glareolus	Finland	
PUUV/Konnevesi_LB2/2008	-	-	JQ319286	2008	Myodes glareolus	Finland	
PUUV/Konnevesi_LB20/2008	-	-	JQ319304	2008	Myodes glareolus	Finland	
PUUV/Konnevesi_LB22/2008	-	-	JQ319306	2008	Myodes glareolus	Finland	
PUUV/Konnevesi_LB25/2008	-	-	JQ319309	2008	Myodes glareolus	Finland	
PUUV/Konnevesi_LB26/2008	-	-	JQ319310	2008	Myodes glareolus	Finland	
PUUV/Konnevesi_LB27/2008	-	-	JQ319311	2008	Myodes	Finland	

Strain Name	GenBank Accession No (S segment)	GenBank Accession No (M segment)	GenBank Accession No (L segment)	Sampling Date	GenBank Host	Country	Lineage
					glareolus		
PUUV/Konnevesi_LB29/2005	-	-	JQ319313	2005	Myodes glareolus	Finland	
PUUV/Konnevesi_LB32/2005	-	-	JQ319316	2005	Myodes glareolus	Finland	
PUUV/Konnevesi_LB35/2008	-	-	JQ319319	2008	Myodes glareolus	Finland	
PUUV/Konnevesi/MgM75/2005	-	-	AM980541	2005	Myodes glareolus	Finland	
PUUV/Konnevesi/MgM105A/2005	-	JQ319173	-	2005	Myodes glareolus	Finland	
PUUV/Konnevesi/MgM110/2005	-	-	AM980545	2005	Myodes glareolus	Finland	
PUUV/Konnevesi/Mg_M114B/2005	JQ319171	JQ319175	-	2005	Myodes glareolus	Finland	
PUUV/Konnevesi/Mg_M94A/2005	JQ319163	-	-	2005	Myodes glareolus	Finland	
PUUV/Konnevesi/Mg_O14B/2005	JQ319166	-	-	2005	Myodes glareolus	Finland	
PUUV/Konnevesi/Mg_O15B/2005	JQ319167	-	-	2005	Myodes glareolus	Finland	
PUUV/Konnevesi/Mg_O22B/2005	JQ319168	-	AM980548	2005	Myodes glareolus	Finland	
PUUV/Konnevesi/Mg_O27B/2005	JQ319169	-	-	2005	Myodes glareolus	Finland	
PUUV/Konnevesi/MgO50/2005	-	-	AM980537	2005	Myodes glareolus	Finland	
PUUV/Konnevesi/Mg_O57A/2005	JQ319161	-	AM980542	2005	Myodes glareolus	Finland	
PUUV/Konnevesi/Mg_O6B/2005	JQ319164	-	-	2005	Myodes glareolus	Finland	
PUUV/Konnevesi/Mg_O74B/2005	JQ319170	-	-	2005	Myodes glareolus	Finland	
PUUV/Konnevesi/Mg_O78A/2005	JQ319162	JQ319172	AM980540	2005	Myodes glareolus	Finland	
PUUV/Konnevesi/Mg_M78B/2005	-	JQ319174	-	2005	Myodes glareolus	Finland	
PUUV/Konnevesi/Mg_O9B/2005	JQ319165	-	-	2005	Myodes glareolus	Finland	
PUUV/Pieksamaki/human_kidney/2008	JN831950	JN831951	-	2008	Human	Finland	

Strain Name	GenBank Accession No (S segment)	GenBank Accession No (M segment)	GenBank Accession No (L segment)	Sampling Date	GenBank Host	Country	Lineage
PUUV/Pieksamaki/human_lung/2008	JN831947	-	-	2008	Human	Finland	
PUUV/Pieksamaki/Mg7/2008	JN831943	JN831944	JN831945	2008	Myodes glareolus	Finland	
Sotkamo	-	NC005223	NC005225	?	Myodes glareolus	Finland	
Sotkamo 2009	HE801633	HE801634	-	2009	Myodes glareolus	Finland	
PUUV/Jelgava/Mg149/2008	JN657228	-	JN657236	2008	Myodes glareolus	Latvia	LAT
KS13/855	-	KF906513	-	2009	Myodes glareolus	Poland	
Aijajarvi/Mg7/05	GQ339476	-	-	2005	Myodes glareolus	Sweden	
Aijajarvi/Mg9/05	GQ339477	-	-	2005	Myodes glareolus	Sweden	
Bussjo_95-1	AM746297	-	-	1995	Myodes glareolus	Sweden	
Bussjo_95-2	AM746298	-	-	1995	Myodes glareolus	Sweden	
Bussjo_95-3	AM746299	-	-	1995	Myodes glareolus	Sweden	
Bussjo_98-1	AM746300	-	-	1998	Myodes glareolus	Sweden	
Bussjo_98-2	AM746301	-	-	1998	Myodes glareolus	Sweden	
Bussjo_98-3	AM746302	-	-	1998	Myodes glareolus	Sweden	N-SCA
Bussjo_98-4	AM746303	-	-	1998	Myodes glareolus	Sweden	
Bussjo_98-5	AM746304	-	-	1998	Myodes glareolus	Sweden	
Bussjo_98-6	AM746305	-	-	1998	Myodes glareolus	Sweden	
Bussjo_98-7	AM746306	-	-	1998	Myodes glareolus	Sweden	
Bussjo_98-8	AM746307	-	-	1998	Myodes glareolus	Sweden	
Bussjo_99-1	AM746308	-	-	1999	Myodes glareolus	Sweden	
Bussjo_99-2	AM746309	-	-	1999	Myodes glareolus	Sweden	

Strain Name	GenBank Accession No (S segment)	GenBank Accession No (M segment)	GenBank Accession No (L segment)	Sampling Date	GenBank Host	Country	Lineage
Djaknebole_98-1	AM746310	-	-	1998	Myodes glareolus	Sweden	
Djaknebole_98-2	AM746311	-	-	1998	Myodes glareolus	Sweden	
Djaknebole_98-3	AM746312	-	-	1998	Myodes glareolus	Sweden	
Djaknebole_98-4	AM746313	-	-	1998	Myodes glareolus	Sweden	
Djaknebole_98-5	AM746314	-	-	1998	Myodes glareolus	Sweden	
Gumboda_98-1	AM746315	-	-	1998	Myodes glareolus	Sweden	
Gumboda_98-2	AM746316	-	-	1998	Myodes glareolus	Sweden	
Gumboda_98-3	AM746317	-	-	1998	Myodes glareolus	Sweden	
Gumboda_98-4	AM746318	-	-	1998	Myodes glareolus	Sweden	
Gumboda_98-5	AM746319	-	-	1998	Myodes glareolus	Sweden	
Gyttjea/Mg19/05	GQ339480	-	-	2005	Myodes glareolus	Sweden	
Jockfall/Mg12/05	GQ339478	-	-	2005	Myodes glareolus	Sweden	
Kalvudden/Mg22/05	GQ339482	-	-	2005	Myodes glareolus	Sweden	
Kiviniemi/Mg3/05	GQ339473	-	-	2005	Myodes glareolus	Sweden	
Kiviniemi/Mg5/05	GQ339474	-	-	2005	Myodes glareolus	Sweden	
Kiviniemi/Mg6/05	GQ339475	-	-	2005	Myodes glareolus	Sweden	
Ljustrask/Mg20/05	GQ339481	-	-	2005	Myodes glareolus	Sweden	
Moskosel/Mg17/05	GQ339479	-	-	2005	Myodes glareolus	Sweden	
Norum_98-1	AM746320	-	-	1998	Myodes glareolus	Sweden	
Norum_98-2	AM746321	-	-	1998	Myodes glareolus	Sweden	
Norum_98-3	AM746322	-	-	1998	Myodes	Sweden	

Strain Name	GenBank Accession No (S segment)	GenBank Accession No (M segment)	GenBank Accession No (L segment)	Sampling Date	GenBank Host	Country	Lineage
					glareolus		
Norum_98-4	AM746323	-	-	1998	Myodes glareolus	Sweden	
Norum_98-5	AM746324	-	-	1998	Myodes glareolus	Sweden	
Norum_98-6	AM746325	-	-	1998	Myodes glareolus	Sweden	
Norum_98-7	AM746326	-	-	1998	Myodes glareolus	Sweden	
Palbole_98-1	AM746327	-	-	1998	Myodes glareolus	Sweden	
Palbole_98-2	AM746328	-	-	1998	Myodes glareolus	Sweden	
Palbole_98-3	AM746329	-	-	1998	Myodes glareolus	Sweden	
Palbole_98-4	AM746330	-	-	1998	Myodes glareolus	Sweden	
Puu/Huggberget/Cg36/94	AJ223371	-	-	1994	Myodes glareolus	Sweden	
Puu/Mellansel/Cg47/94	AJ223374	-	-	1994	Myodes glareolus	Sweden	
Puu/Mellansel/Cg49/94	AJ223375	-	-	1994	Myodes glareolus	Sweden	
Puu/Tavelsjo/Cg81/94	AJ223380	-	-	1994	Myodes glareolus	Sweden	
Puu/Vindeln/L20Cg/83	Z48586	Z49214	-	1983	Myodes glareolus	Sweden	
Skaran_98-1	AM746331	-	-	1998	Myodes glareolus	Sweden	
Skaran_98-2	AM746332	-	-	1998	Myodes glareolus	Sweden	
Skaran_98-3	AM746333	-	-	1998	Myodes glareolus	Sweden	
Umea/hu	AY526219	AY526218	AY526217	1985	Human	Sweden	
Vranica	U14137	U14136	-	1984	Myodes glareolus	Bosnia-Hercegovina	
Baltic/205Cg/00	AJ314599	-	-	2000	Myodes glareolus	Estonia	RUS
Baltic/49Cg/00	AJ314598	-	-	2000	Myodes glareolus	Estonia	

Strain Name	GenBank Accession No (S segment)	GenBank Accession No (M segment)	GenBank Accession No (L segment)	Sampling Date	GenBank Host	Country	Lineage
CG17/Baskiria-2001	AF442613	AF442614	-	2001	Myodes glareolus	Russia	
CG1820	M32750	M29979	M63194	1984	Myodes glareolus	Russia	
DTK/Ufa-97	AB297665	AB297666	AB297667	1997	Myodes glareolus	Russia	
K27	L08804	L08754	-	?	Human	Russia	
P360	L11347	L08755	-	?	Human	Russia	
Kazan	Z84204	Z84205	EF405801	?	Myodes glareolus	Russia	
PUUV/Jelgava/Mg136/2008	JN657230	-	JN657238	2008	Myodes glareolus	Latvia	
PUUV/Jelgava/Mg140/2008	JN657231	-	-	2008	Myodes glareolus	Latvia	
PUUV/Madona/Mg233/2008	JN657232	-	JN657239	2008	Myodes glareolus	Latvia	
PUUV/Madona/Mg99/2008	JN657229	-	JN657237	2008	Myodes glareolus	Latvia	
Samara_112/CG/2005	AB433846	-	-	2005	Myodes glareolus	Russia	
Samara_128/CG/2005	AB433847	-	-	2005	Myodes glareolus	Russia	
Samara_147/CG/2005	AB433848	-	-	2005	Myodes glareolus	Russia	
Samara_49/CG/2005	AB433843	AB433850	AB574183	2005	Myodes glareolus	Russia	
Samara_6/CG/2005	AB433842	-	-	2005	Myodes glareolus	Russia	
Samara_68/CG/2005	AB433844	AB433851	-	2005	Myodes glareolus	Russia	
Samara_94/CG/2005	AB433845	AB433852	AB574184	2005	Myodes glareolus	Russia	
Udmurtia/338Cg/92	Z30708	-	-	1992	Myodes glareolus	Russia	
Udmurtia/444Cg/88	Z30706	-	-	1988	Myodes glareolus	Russia	
Udmurtia/458Cg/88	Z30707	-	-	1988	Myodes glareolus	Russia	
Udmurtia/894Cg/91	Z21497	-	-	1991	Myodes glareolus	Russia	
Bergsjobo/Mg25/05	GQ339483	-	-	2005	Myodes	Sweden	S-SCA

Strain Name	GenBank Accession No (S segment)	GenBank Accession No (M segment)	GenBank Accession No (L segment)	Sampling Date	GenBank Host	Country	Lineage
					glareolus		
Faboviken/Mg26/05	GQ339484	-	-	2005	Myodes glareolus	Sweden	
Mangelbo/Mg1/05	GQ339485	-	-	2005	Myodes glareolus	Sweden	
Munga/Mg16/05	GQ339487	-	-	2005	Myodes glareolus	Sweden	
Munga/Mg2/05	GQ339486	-	-	2005	Myodes glareolus	Sweden	
Puu/Eidsvoll/1124v	AJ223368	-	-	1987	Myodes glareolus	Norway	
Puu/Eidsvoll/Cg1138/87	AJ223369	-	-	1987	Myodes glareolus	Norway	
Puu/Solleftea/Cg3/95	AJ223376	-	-	1995	Myodes glareolus	Sweden	
Puu/Solleftea/Cg6/95	AJ223377	-	-	1995	Myodes glareolus	Sweden	
00-18	DQ138128	-	-	2000	Myodes regulus	South Korea	
11-1	JX028273	JX028272	JX028271	2011	Myodes regulus	South Korea	
11-4	JX046484	JX046483	JX046482	2011	Myodes regulus	South Korea	
11-5	JX046487	JX046486	-	2011	Myodes regulus	South Korea	MUJV
96-1	DQ138133	-	-	1996	Myodes regulus	South Korea	
99-27	DQ138140	-	-	1999	Myodes regulus	South Korea	
99-28	DQ138142	-	-	1999	Myodes regulus	South Korea	
Fusong-Cr-247	EF442087	-	-	2002-2003	Myodes rufocanus	China	FUSV
Fusong-Cr-275	EF442091	-	-	2002-2003	Myodes rufocanus	China	
Kamiiso-8Cr-95	AB010730	-	-	1995	Myodes rufocanus	Japan	
Tobetsu-60Cr-93	AB010731	-	-	1993	Myodes rufocanus	Japan	HOKV
Tobetsu27S/2004	AB675465	-	-	2004	Myodes rufocanus	Japan	

Strain Name	GenBank Accession No (S segment)	GenBank Accession No (M segment)	GenBank Accession No (L segment)	Sampling Date	GenBank Host	Country	Lineage
Tobetsu35S/2010	AB675450	-	-	2010	Myodes rufocanus	Japan	

© 2015 by the authors; licensee MDPI, Basel, Switzerland. This article is an open access article distributed under the terms and conditions of the Creative Commons by Attribution (CC-BY) license (http://creativecommons.org/licenses/by/4.0/).
